# Effects of chrysin (5,7-dihydroxyflavone) on vascular remodeling in hypoxia-induced pulmonary hypertension in rats

**DOI:** 10.1186/s13020-015-0032-2

**Published:** 2015-02-18

**Authors:** Xian-Wei Li, Xiang-Ming Wang, Shu Li, Jie-Ren Yang

**Affiliations:** Department of Pharmacology, Wannan Medical College, Anhui, 241002 China; Department of Pathology, Yijishan Hospital, Wannan Medical College, Anhui, 241002 China; Department of Pathophysiology, Wannan Medical College, Anhui, 241002 China

**Keywords:** Chrysin, Pulmonary hypertension, NOX4, Reactive oxygen species, Collagen

## Abstract

**Background:**

Chrysin (5,7-dihydroxyflavone) inhibits platelet-derived growth factor-induced vascular smooth muscle cell proliferation and arterial intima hyperplasia. This study aims to investigate the effects of chrysin on rat pulmonary vascular remodeling in hypoxia-induced pulmonary hypertension (PH).

**Methods:**

Sprague–Dawley rats were continuously exposed to 10% O_2_ for 4 weeks to induce PH. The effect of chrysin (50 or 100 mg/kg/d, subcutaneous) on vascular remodeling was investigated in hypoxia-induced PH model. At the end of the experiments, the indexes for pulmonary vascular remodeling and right ventricle hypertrophy were measured by vascular medial wall thickness and the ratio of right ventricle to (left ventricle plus septum). The expressions of NOX4, collagen I, and collagen III were analyzed by immunohistochemistry, real-time PCR, or western blotting. The proliferation of cultured pulmonary artery smooth muscle cells (PASMCs) was determined by BrdU incorporation and flow cytometry. The levels of malondialdehyde (MDA) and reactive oxygen species (ROS) were also determined by thiobarbituric acid reactive substances assay and 2′7′-dichlorofluorescein diacetate method.

**Results:**

Chrysin treatment for 4 weeks significantly attenuated pulmonary vascular remodeling and improved collagen accumulation and down-regulated collagen I and collagen III expressions, accompanied by downregulation of NOX4 expression in the pulmonary artery (*P* = 0.012 for 50 mg/kg/d, *P* < 0.001 for 100 mg/kg/d) and lung tissue (*P* = 0.026, *P* < 0.001). *In vitro*, chrysin (1, 10, and 100 μM) remarkably attenuated PASMC proliferation (*P* = 0.021 for 1 μM, *P* < 0.001 for 10 μM, and *P* < 0.001 for 100 μM), collagen I expression (*P* = 0.035, *P* < 0.001, and *P* < 0.001), and collagen III expression (*P* = 0.027, *P* < 0.001, and *P* < 0.001) induced by hypoxia, and these inhibitory effects of chrysin were accompanied by inhibition of NOX4 expression (*P* = 0.019, *P* < 0.001, and *P* < 0.001), ROS production (*P* = 0.038, *P* < 0.001, and *P* < 0.001), and MDA generation (*P* = 0.024, *P* < 0.001, and *P* < 0.001).

**Conclusions:**

This study demonstrated that chrysin treatment in hypoxia-induced PH in rats reversed the hypoxia-induced (1) elevations of NOX4 expression, (2) productions of ROS and MDA, (3) proliferation of PASMC, and (4) accumulation of collagen.

## Background

Pulmonary hypertension (PH) is a syndrome in which obstructed, constricted small pulmonary arteries and increased pulmonary vascular resistance ultimately lead to right ventricular hypertrophy and failure [1]. Although the pathogenesis of PH has not been fully understood, it is well-accepted that vascular remodeling is a hallmark of PH [2,3]. The proliferation and migration of pulmonary artery smooth muscle cells (PASMCs) and accumulation of extracellular matrix (ECM) components, such as collagens, are important processes in pulmonary vascular structural remodeling [4-6]. Current therapies for chronic PH-induced vasodilation reduce pulmonary arterial resistance (*e.g.*, nitric oxide inhalation, stimulation of cGMP production by phosphodiesterase inhibitors, endothelin receptor antagonists, and prostacyclin analogs) [7]. Oxidative stress is suggested to contribute greatly to the development of PH and antioxidant therapy might be a novel strategy for PH [8,9].

Agents promoting reactive oxygen species (ROS) generation stimulate both systemic arterial smooth muscle cells and PASMC proliferation [10], implicating ROS in the vascular remodeling associated with chronic hypoxia. Meanwhile, suppression of endogenous ROS production inhibits smooth muscle cell proliferation and promotes apoptosis [11,12]. In animal models, ROS production has been directly linked to the vascular remodeling associated with chronic hypoxia-induced PH [13,14]. Among the sources of ROS, NADPH oxidase (NOX) can generate ROS in a highly regulated manner. Mice maintained for 21 days under hypoxic (10% O_2_) conditions had substantially increased NADPH oxidase homologue NOX4 expression in medial PASMCs [15]. Importantly, pulmonary arteries from subjects with idiopathic pulmonary arterial hypertension also had increased expression of NOX4 [16,17]. Reduction of NOX4 expression by rosiglitazone administration attenuated hypoxia-induced PH and vascular remodeling in mice [18]. Taken together, these reports indicate that NOX4 is an important mediator of PH caused by hypoxia.

Chrysin (5,7-dihydroxyflavone) is a natural flavonoid from many plant extracts, honey, and propolis [19,20], with many biological and pharmacological activities, such as anti-inflammatory, anticancer, antioxidant, and antihypertensive effects [21-25]. The beneficial effects of chrysin on the vascular endothelium was due to nitric oxide released from the endothelium and aortic relaxation [26,27]. Chrysin inhibited the platelet-derived growth factor-induced vascular smooth muscle cell proliferation, and the arterial intima hyperplasia due to its antioxidative effects [28]. This study aims to investigate the effects of chrysin on rat pulmonary vascular remodeling in hypoxia-induced PH.

## Methods

### Materials and reagents

Chrysin (purity: >98%) was obtained from Shanghai Pure-one Bio Technology (China; CAS: 480-40-0). A Masson’s trichrome staining kit was purchased from Nanjing KeyGEN Biotech (China). A BrdU cell proliferation assay kit was provided by Roche (Germany). Dulbecco’s modified Eagle’s medium (DMEM) was provided by GIBCO (USA). Dimethyl sulfoxide (DMSO) and diphenyleneiodonium chloride (DPI; D2926) were purchased from Sigma (USA). Lipid peroxidation malondialdehyde (MDA) assay kit and ROS assay kit were supplied by Beyotime Institute of Biotechnology (China). The primers were purchased from Shanghai Sangon Biological Engineering Co. Ltd. (China). A PrimeScript reverse transcription reagent kit and SYBR® Premix Ex Taq™ were obtained from TaKaRa Biotechnology Co. Ltd. (China). Anti-β-actin, anti-α-actin, anti-NOX4, anti-collagen I, and anti-collagen III primary antibodies, and horseradish peroxidase-conjugated secondary antibodies were purchased from Abcam (Hong Kong). The EasySee Western Blot Kit was provided by Beijing TransGen Biotech (China).

### Animals

Male Sprague–Dawley rats were obtained from Nanjing Qinglongshan Experimental Animal Company (China; Certificate No: SCXK [jun] 2007–012). All experiments were conducted in accordance with the US National Institutes of Health Guide for the Care and Use of Laboratory Animals, and the experimental protocol was approved by the Medicine Ethical Committee of Wannan Medical College (NO.201401, date: 2014-10-13).

### Animal experiments

Rats (*n* = 48; age: 6–8 weeks; weight: 180–220 g) were acclimated for 1 week, and then arbitrarily distributed into four groups: normoxia group; hypoxia group; hypoxia plus chrysin (50 mg/kg/d) group; and hypoxia plus chrysin (100 mg/kg/d) group. Chrysin was initially dissolved in Tris-buffer at pH 8.9, and the pH was then adjusted to 7.2 with 1 N HCl (Beijing institute of chemical reagents,China). Chrysin was administered *via* subcutaneous injection once daily. The rats in the normoxia group and hypoxia group received corresponding injections of saline solution (Qilu Pharmaceutical Co.,LTD,China). The rats in the normoxia group were placed in normoxia (21% O_2_). The rats in the hypoxia group and hypoxia plus chrysin groups were placed in a chamber and continuously exposed to 10% O_2_ for 4 weeks. At the end of the experiment, the animals were anesthetized with sodium pentobarbital (30 mg/kg, intraperitoneal) (Shanghai solarbio Bioscience & Technology Co., LTD, China), and the right ventricular systolic pressure (RVSP) and mean pulmonary artery pressure (mPAP) were monitored. After euthanasia of the animals, the right ventricle (RV), left ventricle (LV), and interventricular septum (S) were dissected from the heart and weighed to calculate the ratio of RV to (LV + S), a key index for evaluating hypertrophy of the RV. The freshly isolated pulmonary arterial samples were used for mRNA and protein expression analyses. The excised lungs were fixed in 4% paraformaldehyde for hematoxylin-eosin and immunohistochemical staining.

### Lung tissue histology and Masson’s trichrome staining

Hematoxylin-eosin staining and Masson’s trichrome staining were performed on paraffin sections (5-μm thickness). For quantification of the pulmonary arterial wall thickness, the lumen diameter (or area at the basement membrane level) and total vascular diameter (or area at the adventitial border) in 10 muscular arteries with diameters of 100–200 μm per lung section were outlined. The percentage of vascular medial wall thickness (WT) was calculated by the following formula:

$$ \mathrm{W}\mathrm{T}\ \left(\%\right) = {\mathrm{area}}_{\mathrm{ext}}\hbox{--} {\mathrm{area}}_{\mathrm{int}}/{\mathrm{area}}_{\mathrm{ext}} \times 100 $$

where area_ext_ and area_int_ are the areas bounded by the external and internal elastic laminae, respectively.

Masson’s trichrome staining was used to demonstrate collagen deposition, in which collagen fibers were stained blue, nuclei were stained dark red/purple, and the cytoplasm was stained red/pink. The procedure was performed according to the manufacturer’s instructions. The collagen volume fraction (CVF) and perivascular collagen area (PVCA) were measured and quantified using Image-Pro plus 6.0 (Media Cybernetics, USA) to evaluate the magnitude of collagen accumulation. The CVF in the interstitial space of the lung tissue was determined by calculating the ratio of the collagen area to the entire area of an individual section (CVF = blue area/(blue area + red area) × 100%). The PVCA was analyzed by calculating the ratio of the fibrotic area (blue area) surrounding the vessel to the total vessel wall area.

### Immunohistochemical assay

For NOX4 immunohistochemical staining in the lung, serial sections of formalin-fixed paraffin-embedded lung tissues were treated with 3% H_2_O_2_ for 20 min at room temperature, followed by preincubation with 10% non-immunized serum. The sections were then incubated with a rabbit anti-NOX4 antibody (1:100) overnight at 4°C. After unbound antibodies were washed off, the sections were incubated with a biotinylated goat anti-rabbit secondary antibody (1:500), and thereafter incubated with streptavidin-horseradish peroxidase. Subsequently, the bound antibodies were visualized by a color reaction with diaminobenzidine as the substrate. After counterstaining with hematoxylin, the sections were dehydrated and permanently mounted. The sections were digitized using an Olympus BX51 microscope (Olympus Optical Co. Ltd., Japan). The digital images were processed by the Image-Pro plus 6.0 software (USA). The positive area and optical density (OD) of NOX4 were determined by measuring three randomly selected microscopic fields (25 × 10) for each slide. The immunohistochemistry index was defined as the average integral optical density (AIOD) (AIOD = positive area × OD/total area).

### Cell experiments

PASMCs were prepared from the pulmonary arteries of male 10-week-old Sprague–Dawley rats by an explant method, as described previously [27,28]. The cells were cultured at 37°C under 5% CO_2_ in DMEM containing 20% fetal bovine serum. PASMCs were identified by immunofluorescence staining with an anti-smooth muscle α-actin (1:50). Cells between passages 3 and 8 were used for experiments. The cells were divided into seven groups to explore the effects of chrysin on hypoxia-induced proliferation of PASMCs, as follows: (i) normoxia: cells were treated with 21% O_2_ for 48 h; (ii) hypoxia, cells were stimulated to proliferate by exposure to hypoxia (3% O_2_, 5% CO_2_, 92% N_2_) for 48 h; (iii) + DMSO: cells were pretreated with DMSO for 1 h, and then subjected (3% O_2_, 5% CO_2_, 92% N_2_) for 48 h; (iv) + DPI (25 μM): cells were pretreated with 25 μM DPI (NADPH oxidase inhibitor) for 1 h, and then subjected to (3% O_2_, 5% CO_2_, 92% N_2_) for 48 h; and (v)–(vii): +chrysin (1, 10, 100 μM): cells were pretreated with chrysin for 1 h, and then subjected to hypoxia (3% O_2_, 5% CO_2_, 92% N_2_) for 48 h. Chrysin and DPI were dissolved in DMSO. Therefore, 0.1% DMSO was used as a vehicle control. In our pilot study, we found that this concentration of DMSO had no effects on cell growth or death.

### Cell proliferation assays

As described in our previous papers [29,30], cell proliferation was measured by two methods. Specifically, DNA synthesis and the cell cycle were analyzed by BrdU marking and flow cytometry, respectively.

### Intracellular ROS production

Intracellular ROS was detected by DCFH-DA. DCFH-DA diffuses into cells and is hydrolyzed into nonfluorescent 2′,7′-dichlorofluorescein (DCFH). Produced ROS, including H_2_O_2_, superoxide, and OH•, oxidize nonfluorescent intracellular DCFH to highly fluorescent dichlorofluorescein (DCF). In this study, cells in 96-well plates at a density of 6 × 10^3^ cells/well were allowed to grow to the desired confluence, and then treated with chrysin (1, 10, or 100 μM) for 1 h prior to hypoxia. Subsequently, the cells were incubated with DCFH-DA in the dark at 37°C for 20 min. DCF fluorescence was detected by a fluorescence microscope and photographs were taken. The relative levels of fluorescence were then quantified in a fluorospectrophotometer (F4000; Hitachi Software Engineering, Yokohama, Japan) with an excitation wavelength of 488 nm and an emission wavelength of 525 nm.

### Determination of MDA concentrations

MDA, a degradation product of lipid peroxidation, is a class of thiobarbituric acid reactive substances (TBARS). The MDA levels were measured using an assay for TBARS, as described previously [31]. The protein concentrations were determined by the bicinchoninic acid assay.

### Real-time PCR analysis

Total RNA was extracted using TRIzol reagent (Invitrogen, USA), and the isolated RNA (0.2–0.5 μg) was reverse-transcribed by a PrimeScript reverse transcription reagent kit. Quantitative analyses of changes in expression levels were performed using SYBR® Premix Ex Taq™ in an ABI 7300 system (Applied Biosystems by Life Technologies, USA). The PCR cycling conditions comprised an initial incubation at 95°C for 15 s, followed by 40 cycles of denaturation at 95°C for 5 s and annealing at 60°C for 31 s. The following primers were used: NOX4: forward, 5′-CCAGAATGAGGATCCCAGAA-3′ and reverse 5′- AGCAGCAGCAGCATGTAGAA-3′; collagen I: forward, 5′-TGCTGCCTTTTCTGTTCCTT-3′ and reverse, 5′-AAGGTGCTGGGTAGGGAAGT-3′; collagen III: forward, 5′-GTCCACGAGGTGACAAAGGT-3′ and reverse, 5′-CATCTTTTCCAGGAGGTCCA-3′; β-actin: forward, 5′-TGTCACCAACTGGGACGATA-3′ and reverse, 5′-ACCCTCATAGATGGGCACAG-3′. Data analyses were performed by the comparative Ct method by the ABI system software. GAPDH was evaluated for normalization of the mRNA expression levels.

### Western blot analysis

Total proteins were extracted from pulmonary arteries, lung tissues, and PASMCs using RIPA buffer containing 0.1% PMSF (Beyotime Institute of Biotechnology, China), and the protein concentrations were determined using the Bradford method. Equal amounts of proteins from each sample (50 μg) were separated by 10% SDS-PAGE and transferred to polyvinylidene fluoride membranes. The membranes were incubated with anti-NOX4 (1:1,000), anti-collagen I (1:500), anti-collagen III (1:500), or anti-β-actin (1:2000) primary antibodies overnight at 4°C, followed by horseradish peroxidase-conjugated goat anti-rabbit (1:2000) or rabbit anti-mouse (1:2000) secondary antibodies for 1 h. The chemiluminescence signals were detected with an EasySee Western Blot Kit. Densitometric analyses were conducted with Image J 1.43 software (National Institutes of Health, USA).

### Statistical analysis

The results were presented as means ± standard deviation (SD). Statistical analyses were performed by ANOVA by SPSS 17.0 (IBM, USA) followed by the Newman–Student–Keuls test for multiple comparisons. Values of *P* < 0.05 were considered significant, and exact *P*-values were shown unless *P* < 0.001.

## Results

### Chrysin treatment ameliorated homodynamic and cardiovascular remodeling

**Figure 1:**
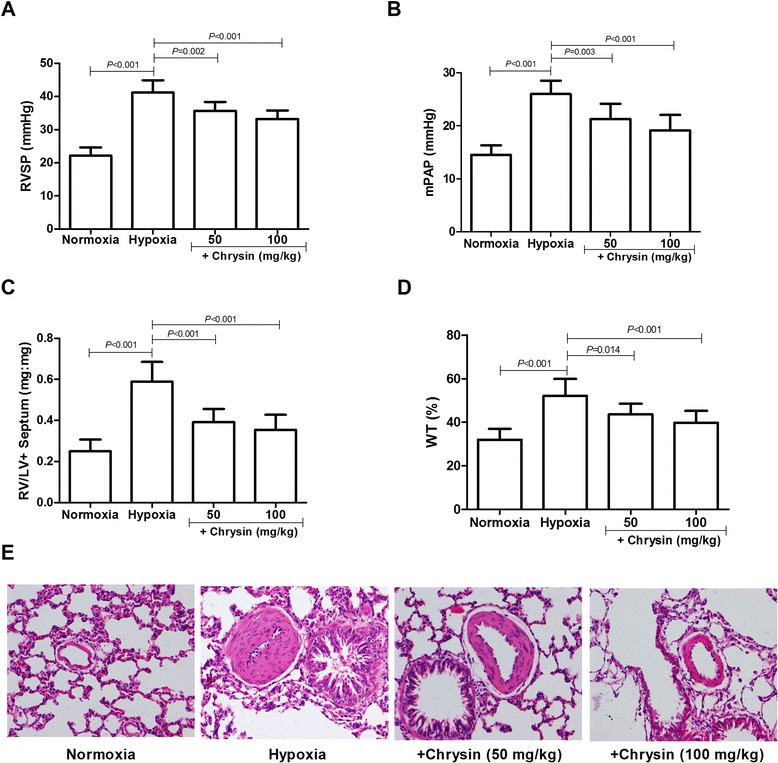
**Effects of chrysin on cardiovascular remodeling in rats with hypoxia-induced PH. (A)** Right ventricular systolic pressure (RVSP) values. **(B)** Mean pulmonary artery pressure (mPAP) values. **(C)** Ratios of right ventricle (RV) weight to weight of the left ventricle (LV) plus interventricular septum (S). **(D)** Statistical graph analyses of pulmonary vascular medial wall thickness (WT). **(E)** Hematoxylin-eosin staining of lung tissues. Data are means ± SD (*n* = 8 rats per group). Statistical analysis was performed with one-way ANOVA followed by the Newman–Student–Keuls test for multiple comparisons.

Consistent with previous studies [30], 4 weeks of exposure to hypoxia induced PH in rats, as shown by significant elevations in RVSP and mPAP compared with the normoxic rats (Figure 1A,B). Hypoxia also induced significant hypertrophy of the RV and pulmonary arteries, with increases in the ratio of RV/LV + S, percentage of WT, and proliferation of smooth muscle cells in the vascular media of small pulmonary arteries compared with the normoxia group (Figure 1C-E). All of these effects of hypoxia were significantly alleviated by treatment of rats with chrysin (50 or 100 mg/kg/d).

### Chrysin treatment decreased collagen accumulation in pulmonary arteries

**Figure 2:**
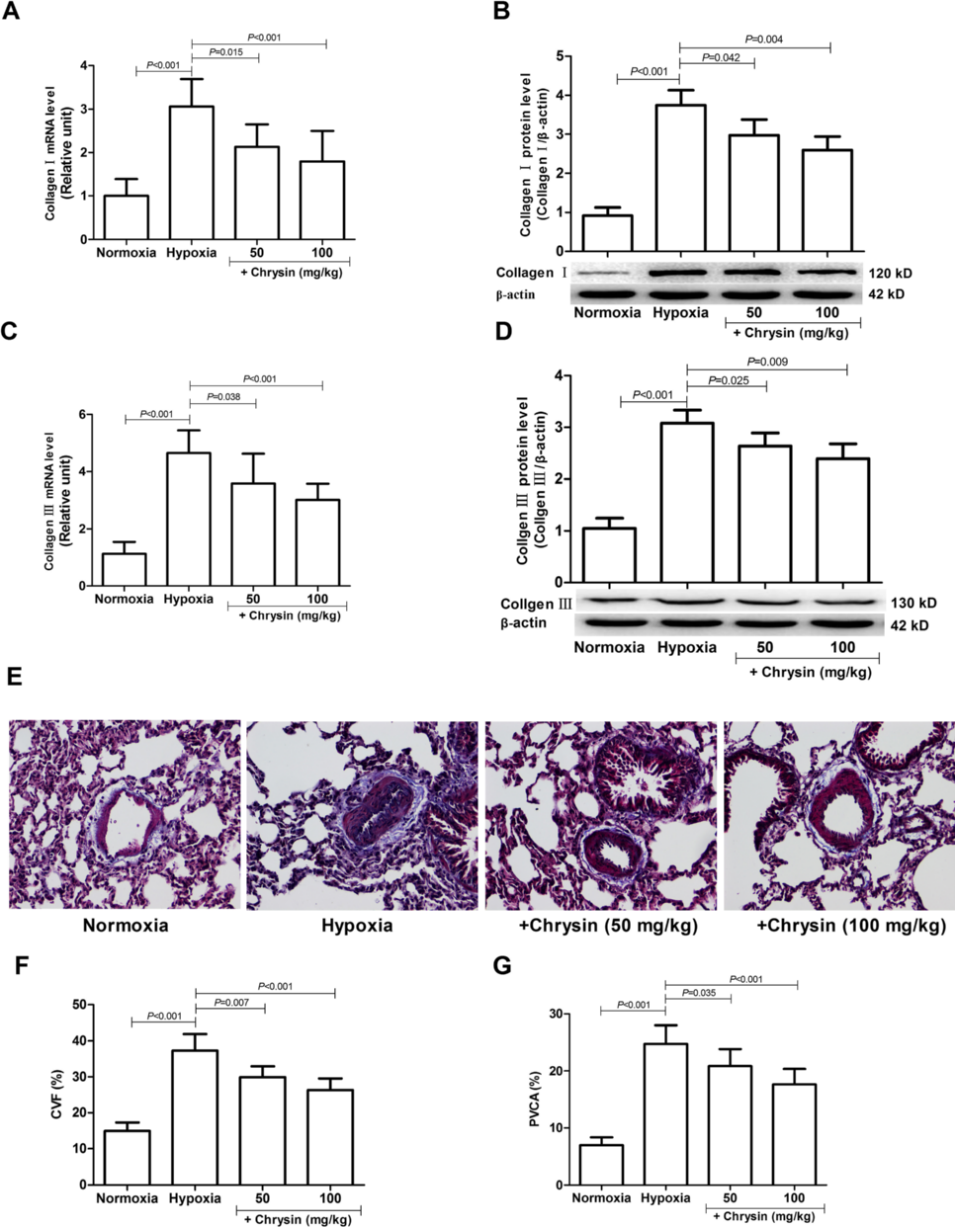
**Chrysin attenuated collagen expressions in pulmonary arteries from rats with hypoxia -induced PH. (A, C)** The expressions of collagen I and collagen III mRNAs were determined by real-time PCR. **(B, D)** The expressions of collagen I and collagen III proteins were determined by western blotting. **(E)** Masson’s trichrome staining of lung tissues. **(F)** Collagen volume fraction (CVF) values of lung tissues. **(G)** Perivascular collagen area (PVCA) values. Data are means ± SD (*n* = 8 rats per group). Statistical analysis was performed with one-way ANOVA followed by the Newman–Student–Keuls test for multiple comparisons.

Hypoxia markedly upregulated the expressions of collagen I and collagen III mRNAs and proteins in pulmonary arteries (Figure 2A-D). Hypoxia also significantly increased the amounts of collagen accumulation in pulmonary arteries evaluated by Masson’s trichrome staining (Figure 2E-G). All of these effects of hypoxia were significantly decreased by treatment of rats with chrysin (50 or 100 mg/kg/d).

### Chrysin inhibited proliferation of PASMCs

**Figure 3:**
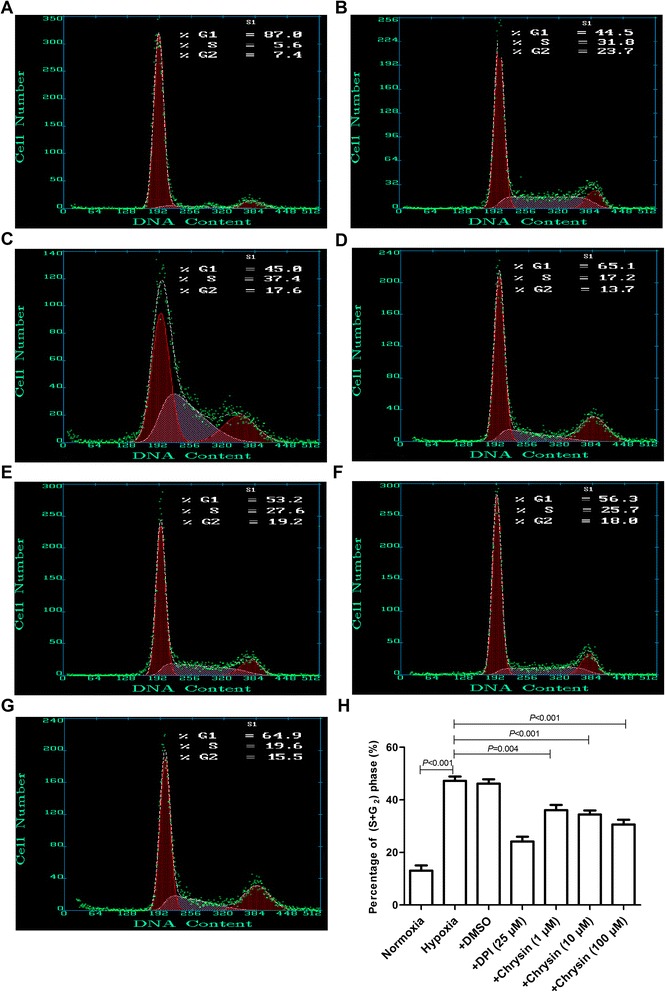
**Effects of chrysin on hypoxia-induced G2/M cell cycle arrest in PASMCs. (A–G)** Cell cycle distribution was monitored by flow cytometry using a propidium iodide staining assay for the following groups: **(A)** Normoxia; **(B)** Hypoxia; **(C)** + DMSO; **(D)** + DPI (25 μM); **(E)**–**(G)** PASMCs pretreated with chrysin (1, 10, and 100 μM) before hypoxia treatment, respectively. **(H)** Percentages of cells in S + G2 phase. Data are means ± SD from three independent experiments. Statistical analysis was performed with one-way ANOVA followed by the Newman–Student–Keuls test for multiple comparisons.DPI: diphenyleneiodonium chloride.

**Figure 4:**
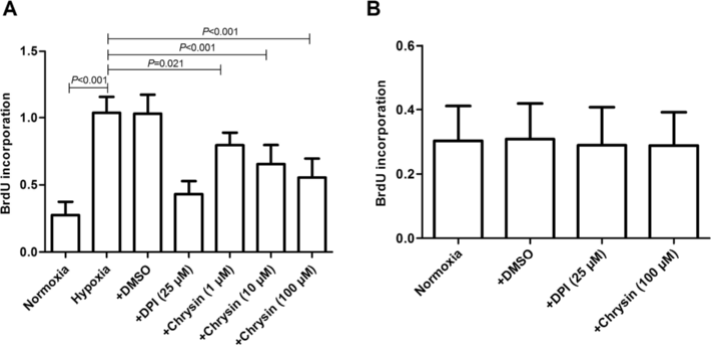
**Chrysin inhibited the proliferation of PASMCs induced by hypoxia. (A, B)** Cell proliferation was measured by BrdU incorporation assays. Data are means ± SD from three independent experiments. Statistical analysis was performed with one-way ANOVA followed by the Newman–Student–Keuls test for multiple comparisons.

Hypoxia stimulated the proliferation of PASMCs, as shown by increases in the percentage of cells in S + G_2_ phase and BrdU incorporation. Treatment with chrysin (1, 10, and 100 μM) and DPI (25 μM) significantly inhibited hypoxia-induced proliferation of PASMCs (Figures 3 and 4A). However, DMSO, DPI, or chrysin (100 μM) alone had no effects on the proliferation of PASMCs under normoxia (Figure 4B).

### Chrysin suppresseed collagen I and collagen III expressions in PASMCs

**Figure 5:**
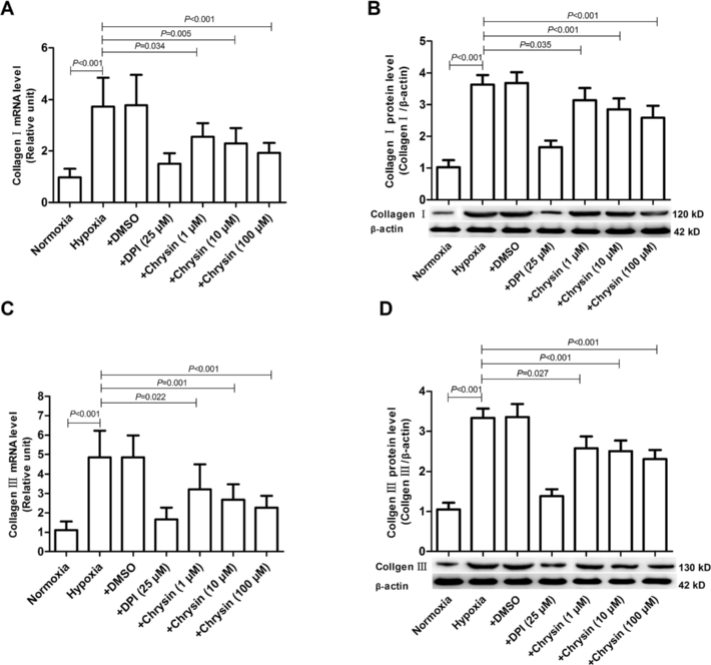
**Effects of chrysin on hypoxia-induced expressions of collagen I and collagen III in PASMCs. (A, C)** The expressions of collagen I and collagen III mRNAs were determined by real-time PCR. **(B, D)** The expressions of collagen I and collagen III proteins were determined by western blotting. Data are means ± SD from three independent experiments. Statistical analysis was performed with one-way ANOVA followed by the Newman–Student–Keuls test for multiple comparisons. DPI: diphenyleneiodonium chloride.

Hypoxia upregulated the expressions of collagen I and collagen III mRNAs and proteins in PASMCs, as evaluated by real-time PCR and western blotting, respectively. Treatment with chrysin (1, 10, and 100 μM) and DPI significantly inhibited the upregulated mRNA and protein expressions of collagen I and collagen III induced by hypoxia (Figure 5).

### Chrysin inhibited NOX4 expression

**Figure 6:**
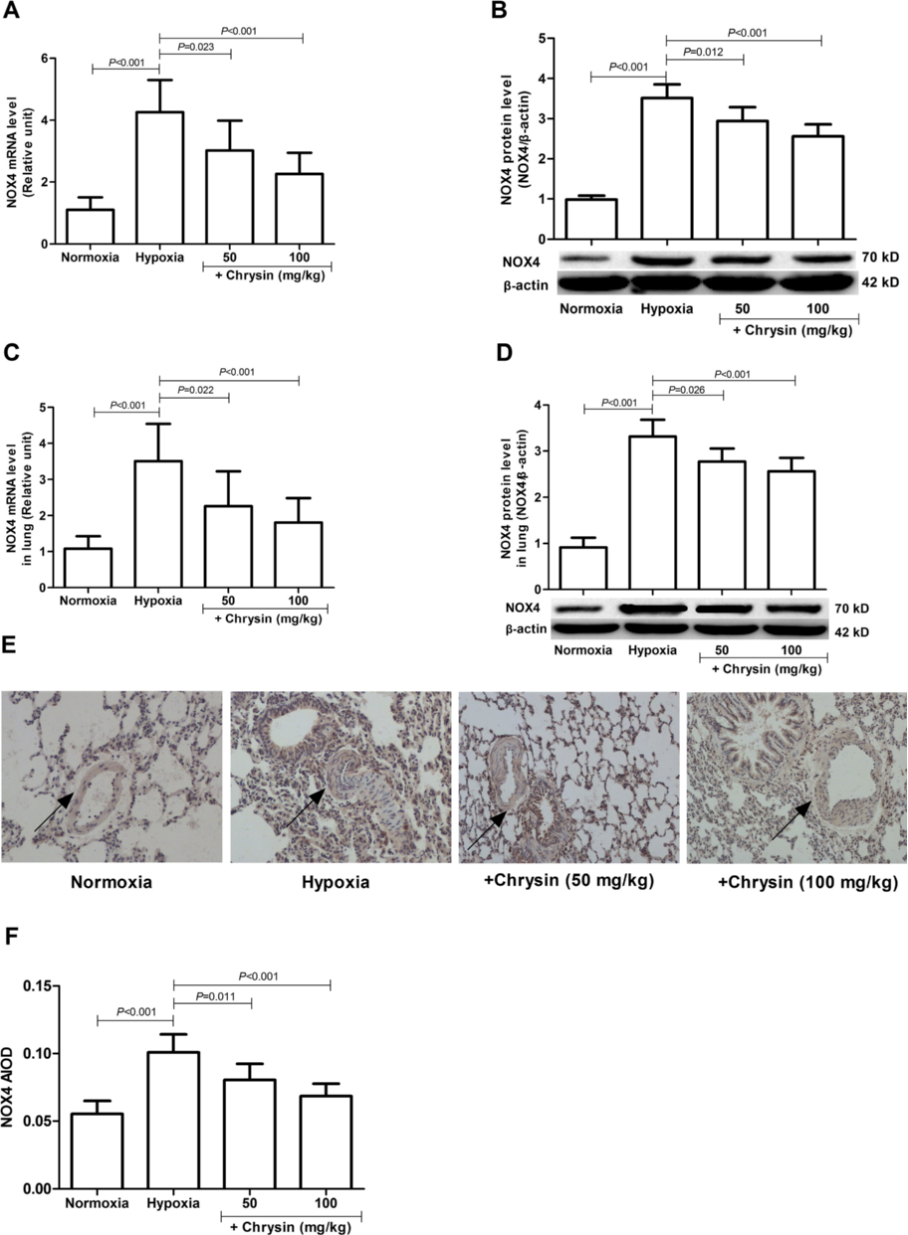
**Effects of chrysin on NOX4 expression in pulmonary arteries and lung tissues from rats with hypoxia-induced PH. (A, B)** The expressions of NOX4 mRNA and protein in pulmonary arteries were determined by real-time PCR and western blotting, respectively. **(C, D)** The expressions of NOX4 mRNA and protein in lung tissues were determined by real-time PCR and western blotting, respectively. **(E)** The expression of NOX4 in lung tissues was determined by immunohistochemical staining (arrows indicate NOX4-positive staining). **(F)** NOX4 average integral optical density (AIOD) values. Data are means ± SD (*n* = 8 rats per group). Statistical analysis was performed with one-way ANOVA followed by the Newman–Student–Keuls test for multiple comparisons. NOX4: NADPH oxidase 4.

**Figure 7:**
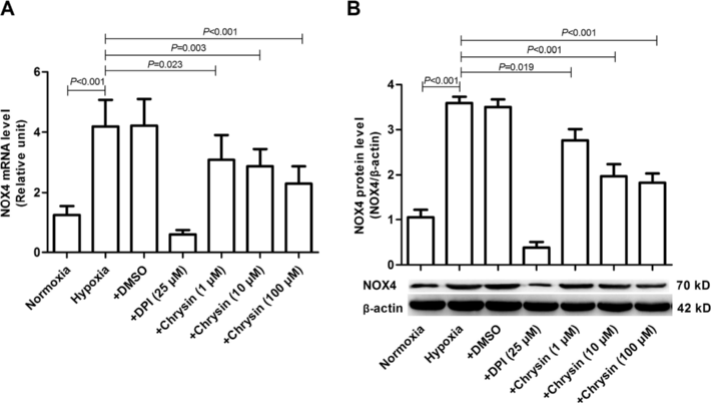
**Chrysin downregulated NOX4 expression induced by hypoxia in cultured PASMCs. (A)** The expression of NOX4 mRNA was determined by real-time PCR. **(B)** The expression of NOX4 protein was determined by western blotting. Data are means ± SD from three independent experiments. Statistical analysis was performed with one-way ANOVA followed by the Newman–Student–Keuls test for multiple comparisons. NOX4: NADPH oxidase 4; DPI: diphenyleneiodonium chloride.

Hypoxia upregulated the expressions of NOX4 mRNA and protein in pulmonary arteries - (Figure 6A,B) and lung tissues- (Figure 6C-F) of rats. Chrysin inhibited the hypoxia-induced upregulated expressions of both NOX4 mRNA and protein -. PASMCs were stimulated with hypoxia in the presence or absence of chrysin (1, 10, and 100 μM) for specified times to investigate whether chrysin could inhibit NOX4 expression directly. Exposure of PASMCs to hypoxia for 48 h significantly increased the mRNA and protein levels of NOX4, while chrysin and DPI significantly inhibited the hypoxia-induced upregulated expressions of both NOX4 mRNA and protein (Figure 7).

### Effects of chrysin on ROS and MDA generation in PASMCs

**Figure 8:**
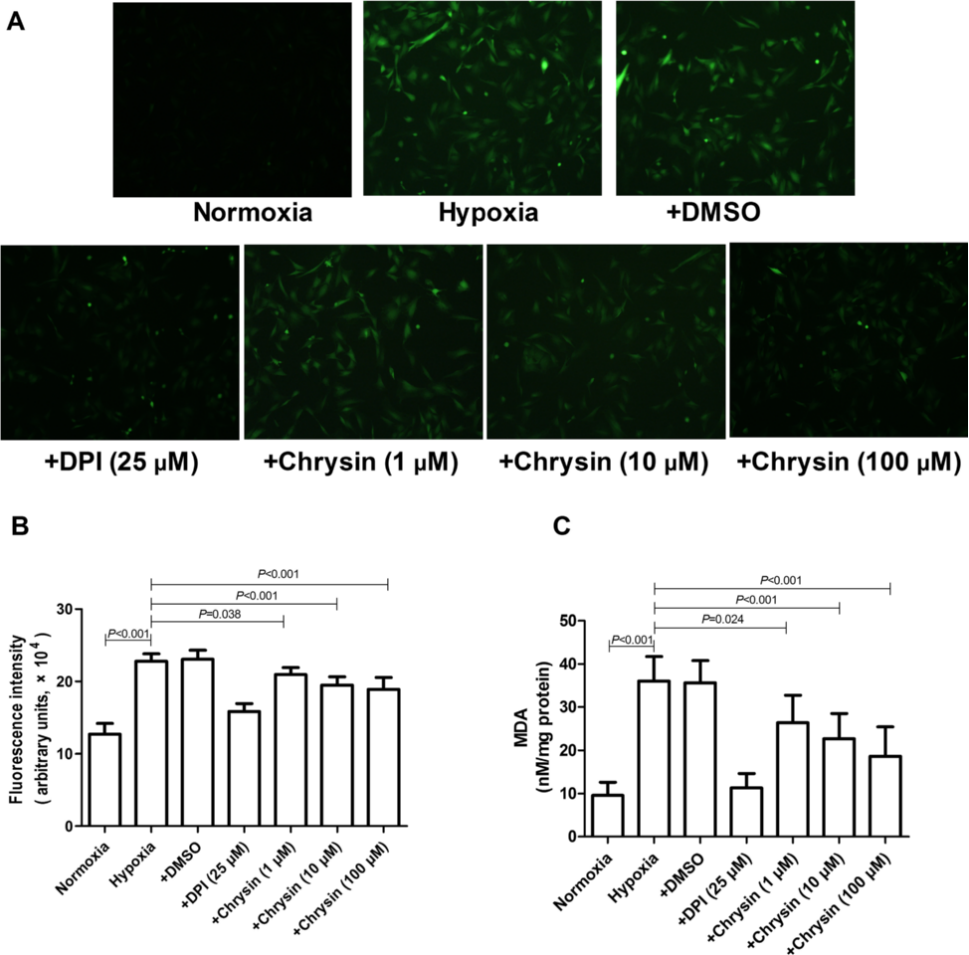
**Effects of chrysin on hypoxia-induced ROS generation in cultured PASMCs. (A)** Representative images of ROS fluorescence in different groups. **(B)** Levels of intracellular ROS, as shown by DCF fluorescence intensities. **(C)** Levels of MDA. Data are means ± SD from three independent experiments. Statistical analysis was performed with one-way ANOVA followed by the Newman–Student–Keuls test for multiple comparisons. ROS: reactive oxygen species; MDA: malondialdehyde; DPI: diphenyleneiodonium chloride.

Exposure of PASMCs to hypoxia for 48 h increased the ROS and MDA productions, while chrysin or DPI significantly reduced the increases in ROS generation and MDA content (Figure 8).

## Discussion

In the present study, homodynamic analyses and histological findings demonstrated that chrysin treatment inhibited the upregulated NOX4 and collagen expressions by hypoxia both *in vivo* and *in vitro*, during the processes of the pulmonary vascular remodeling and process of PH in a rat model. We also found that chrysin significantly inhibited PASMC proliferation and ROS and MDA generations induced by hypoxia *in vitro*.

Chronic hypoxia in the pulmonary vasculature is recognized as an important cause of endothelial dysfunction, PASMC proliferation, and accumulation of ECM components, which lead to vascular remodeling [32]. In keeping with previous study [33,34], on the 28^th^ day of hypoxia, the vessel wall thickness and the ratio of vessel wall thickness to vessel diameter were significantly increased concomitantly with elevated pulmonary arterial pressure and upregulated expressions of collagen I and III. In cultured primary PASMCs, hypoxia exposure induced PASMC proliferation and collagen accumulation, consistent with the *in vivo* findings in rats. In addition, chrysin (50 or 100 mg/kg/d) significantly inhibited the hypertrophy of pulmonary arteries and collagen I and collagen III expressions in hypoxia-induced PH rats, inhibited hypoxia-induced proliferation of PASMCs accompanied by downregulation of collagen I and collagen III expressions. These results suggest a potential for developing chrysin as a drug to treat vascular diseases.

ROS are important regulators of vascular tone and function [35]. In the lung, ROS are implicated in acute hypoxic vasoconstriction [36]. Furthermore, chronic hypoxia-associated increases in ROS generation may interact with and modulate agonist-mediated pulmonary artery vasoconstrictor responses. The sources of ROS in the pulmonary vasculature are not clear. However, there is mounting evidence that NADPH oxidases contribute to systemic vascular pathology [37,38]. These NOX proteins have been implicated in the pathogenesis of pulmonary vascular remodeling.

NOX4-derived ROS mediates hypoxia-induced PASMC proliferation [39], and that hypoxia-induced increases in smooth muscle cells surrounding small pulmonary vessels characterize hypoxia-induced pulmonary vascular alterations in mice [16]. In line with these previous studies, the present study found that hypoxia stimulated NOX4 expression, ROS and MDA productions, and PASMC proliferation. In addition, chrysin significantly attenuated hypoxia-induced NOX4 expression, and ROS and MDA productions concomitantly with decreased PASMC proliferation, suggesting that inhibition of NOX4-derived ROS production might contribute to the protective effects of chrysin against PASMC proliferation and pulmonary vascular remodeling. However, it has also been documented that the NADPH oxidase subunit NOX2 was involved in the development of hypoxia-induced PH [39]. The issue of whether NOX2 was involved in the role of chrysin in the pathogenesis of PH under our settings warrants further investigation.

## Conclusions

This study demonstrated that chrysin treatment in hypoxia-induced PH in rats reversed the hypoxia-induced (1) elevations of NOX4 expression, (2) productions of ROS and MDA, (3) proliferation of PASMC, and (4) accumulation of collagen.

## Abbreviations

PH: Pulmonary hypertension
PASMCs: Pulmonary artery smooth muscle cells
ECM: Extracellular matrix
ROS: Reactive oxygen species
NOX4: NADPH oxidase 4
DCFH-DA: Dichlorofluorescein diacetate
MDA: Malondialdehyde
DMEM: Dulbecco’s modified Eagle’s medium
RVSP: Right ventricle systolic pressure
mPAP: Mean pulmonary artery pressure
WT: Wall thickness
DMSO: Dimethyl sulfoxide
TBARS: Thiobarbituric acid reactive substances
